# Nucleic Acid Amplification Tests for Diagnosis of Smear-Negative TB in a High HIV-Prevalence Setting: A Prospective Cohort Study

**DOI:** 10.1371/journal.pone.0016321

**Published:** 2011-01-27

**Authors:** J. Lucian Davis, Laurence Huang, William Worodria, Henry Masur, Adithya Cattamanchi, Charles Huber, Cecily Miller, Patricia S. Conville, Patrick Murray, Joseph A. Kovacs

**Affiliations:** 1 Division of Pulmonary & Critical Care Medicine, Department of Medicine, San Francisco General Hospital, University of California San Francisco, San Francisco, California, United States of America; 2 HIV/AIDS Division, Department of Medicine, San Francisco General Hospital, University of California San Francisco, San Francisco, California, United States of America; 3 Francis J. Curry International Tuberculosis Center, Department of Medicine, San Francisco General Hospital, University of California San Francisco, San Francisco, California, United States of America; 4 Critical Care Medicine Department, National Institutes of Health Clinical Center, Bethesda, Maryland, United States of America; 5 Department of Laboratory Medicine, National Institutes of Health Clinical Center, Bethesda, Maryland, United States of America; 6 Department of Medicine, Mulago Hospital, Makerere University, Kampala, Uganda; 7 Makerere University-University of California San Francisco Research Collaboration, Kampala, Uganda; University of Cape Town, South Africa

## Abstract

**Background:**

Nucleic acid amplification tests are sensitive for identifying Mycobacterium tuberculosis in populations with positive sputum smears for acid-fast bacilli, but less sensitive in sputum-smear-negative populations. Few studies have evaluated the clinical impact of these tests in low-income countries with high burdens of TB and HIV.

**Methods:**

We prospectively enrolled 211 consecutive adults with cough ≥2 weeks and negative sputum smears at Mulago Hospital in Kampala, Uganda. We tested a single early-morning sputum specimen for Mycobacterium tuberculosis DNA using two nucleic acid amplification tests: a novel in-house polymerase chain reaction targeting the mycobacterial secA1 gene, and the commercial Amplified® Mycobacterium tuberculosis Direct (MTD) test (Gen-Probe Inc, San Diego, CA). We calculated the diagnostic accuracy of these index tests in reference to a primary microbiologic gold standard (positive mycobacterial culture of sputum or bronchoalveolar lavage fluid), and measured their likely clinical impact on additional tuberculosis cases detected among those not prescribed initial TB treatment.

**Results:**

Of 211 patients enrolled, 170 (81%) were HIV-seropositive, with median CD4+ T-cell count 78 cells/µL (interquartile range 29-203). Among HIV-seropositive patients, 94 (55%) reported taking co-trimoxazole prophylaxis and 29 (17%) reported taking antiretroviral therapy. Seventy-five patients (36%) had culture-confirmed TB. Sensitivity of MTD was 39% (95% CI 28–51) and that of secA1 was 24% (95% CI 15–35). Both tests had specificities of 95% (95% CI 90–98). The MTD test correctly identified 18 (24%) TB patients not treated at discharge and led to a 72% relative increase in the smear-negative case detection rate.

**Conclusions:**

The secA1 and MTD nucleic acid amplification tests had moderate sensitivity and high specificity for TB in a predominantly HIV-seropositive population with negative sputum smears. Although newer, more sensitive nucleic acid assays may enhance detection of Mycobacterium tuberculosis in sputum, even currently available tests can provide substantial clinical impact in smear-negative populations.

## Introduction

In 2009, 43% of the 4.6 million new pulmonary tuberculosis cases reported to the World Health Organization were diagnosed without microbiologic confirmation [1]. The failure to confirm a diagnosis with microbiologic testing can result in inappropriate management due to misdiagnosis and failure to initiate appropriate therapy early in the disease process. These disadvantages are especially relevant in low-income countries where supplementary imaging and laboratory data are usually not available to support an empiric diagnosis, and where late presentation for care is more common. A timely and accurate diagnosis of TB must be made if treatment is to be successful and person-to-person transmission reduced. Given the logistical challenges of making reliable sputum smear microscopy and culture facilities widely available, a simple, highly accurate, and rapid diagnostic test could have a dramatic impact on worldwide TB transmission, morbidity, and mortality [2].

Nucleic-acid amplification tests (NAATs) targeting Mycobacterium tuberculosis (MTB) have enormous potential to improve TB case detection, with commercial NAATs, such as the GenProbe Amplified® Mycobacterium Tuberculosis Direct (MTD) Test (Gen-Probe Inc, San Diego, CA), reported to have nearly perfect sensitivity in sputum smear-positive patients and a sensitivity of 61–76% in smear-negative patients [3], [4], [5], [6]. We have previously shown in a small study that a novel NAAT targeting a conserved region of the mycobacterial-genus secA1 gene and capable of identifying all clinically significant species of mycobacteria including M. tuberculosis, has a sensitivity of 100% and a specificity of 90% in sputum smear-negative TB suspects in Uganda, most of whom were HIV-seropositive [7]. In the current study, we evaluated the diagnostic accuracy, incremental value, and potential clinical impact of the secA1 and MTD NAATs in a population at high risk for both HIV and TB.

## Materials and Methods

### Ethics Statement

The Makerere University Faculty of Medicine Research Ethics Committee, the Mulago Hospital Institutional Review Board, the Uganda National Council for Science and Technology, and the University of California San Francisco Committee on Human Research approved the protocol. Some of these patients have been previously included in published studies [8], [9], [10], [11], [12], [13], [14], [15].

### Participants

Between September 2007 and May 2008, we prospectively enrolled consecutive inpatients ≥18 years of age who were admitted to Mulago Hospital in Kampala, Uganda, with cough ≥2 weeks duration into a protocol designed to provide a detailed evaluation (including HIV testing, chest radiography, sputum examination, and bronchoscopy) to identify the etiology of each patient's pulmonary symptoms. Patients with a prior history of TB within two years and those receiving treatment for active TB at the time of hospitalization were excluded. For the current analysis, we included only patients without a positive sputum smear examination by the Ziehl-Neelsen technique, which was the standard method for smear examination at the time of the study.

### Patient Data and Specimen Collection

After obtaining written informed consent from participants, study medical officers recorded demographic and clinical information following a standardized interview, physical exam, and frontal chest radiography. A laboratory technician gave standardized instructions on proper sputum submission [16], after which each patient provided two sputum samples (one spot sample on day one and one early-morning sample on day two) for microscopic examination and culture. The technician recorded the volume and quality of sputum according to technical guidelines [17]. Study medical officers referred HIV-seropositive patients with negative acid-fast bacilli (AFB) smears for bronchoscopy with bronchoalveolar lavage (BAL). Chest physicians performed bronchoscopy according to a standardized protocol that included airway inspection for Kaposi's sarcoma lesions and collection of BAL fluid for microbiologic examination including mycobacterial smear and culture. Full details of bronchoscopy and examination of the clinical specimens have been previously described [11].

At discharge, we asked patients to return in two months for a follow-up clinical evaluation and repeat sputum examination if still symptomatic. After participants had completed all study procedures and the two-month follow-up visit, at least two pulmonary physicians reviewed all available clinical and microbiologic data, and assigned final diagnoses according to explicit clinical definitions as previously described [11].

### Specimen Testing by Smear Microscopy and Mycobacterial Culture

At enrollment, National TB Reference Lab (NTRL) staff examined sputum specimens by direct Ziehl-Neelsen light microscopy and NALC-NaOH concentrated auramine-O fluorescence microscopy using a mercury-vapor lamp, and recorded results semi-quantitatively. In addition, they inoculated Lowenstein-Jensen solid-culture media with concentrated sediment from every sputum and BAL fluid sample collected. Technicians considered cultures positive for MTB when mycobacterial growth of ≥1 colony-forming unit (CFU) was observed within eight weeks of incubation. Positive cultures were confirmed by Ziehl-Neelsen staining, and the amount of growth recorded semi-quantitatively [18].

### Specimen Nucleic Acid Amplification Testing

One-third of the concentrated early-morning sputum pellet remaining after preparation of the microscopy slides and culture slants was frozen and shipped to the National Institutes of Health (NIH) Clinical Center, Bethesda, MD. There it was thawed and divided into two aliquots, which were then randomly labeled and assayed independently in a blinded manner. One aliquot was tested with an in-house polymerase chain reaction (PCR) assay targeting the MTB gene coding for the protein SecA1 as previously described [7], [19]. Briefly, specimens were processed for DNA extraction by ultrasonication for 15 minutes with zirconia/silica beads (BioSpec Products, Bartlesville, OK) in a Tris-HCl buffer containing n-acetyl-cysteine and sodium-dodecyl-sulfate, and then placed in NucliSens lysis buffer (Biomerieux, Boxtel, The Netherlands). DNA was purified using a QIAamp DNA Blood Mini Kit (Qiagen, Germantown, MD). Real-time PCR was performed on a LightCycler (Roche, Basel, Switzerland) using primers targeting a 490-nucleotide region of the secA1 gene [19]. PCR amplification products were detected using fluorescence-resonance energy-transfer (FRET) probes specific for M. tuberculosis complex. Two PCR assays for each sample were performed in separate capillary tubes, one of which was spiked with a plasmid containing an internal-control sequence to allow detection of PCR inhibition. Samples that showed inhibition were repeated using 1∶3 to 1∶100 dilutions as necessary to overcome inhibition. For each PCR run, a negative control (without template DNA) was run to detect contamination. Additional procedures to avoid DNA carry-over were utilized as previously described [7].

The second aliquot was assayed per the manufacturer's protocol using the Gen-Probe Amplified MTD test, which makes multiple copies of a specific MTB 16S-ribosomal RNA sequence for detection by hybridization with a chemiluminescent probe. If a specimen had a chemiluminescence of <30,000 relative light units, it was considered negative, while a positive sample was required to be ≥500,000 units. Specimens between 30,000 units and 499,999 units were re-run, and if the repeat sample was again ≥30,000 units, it was considered positive, as per the manufacturer's protocol. For both secA1 PCR and the MTD assay, a positive control (M. tuberculosis) and a negative control (M. gordonae) were included with each run.

Laboratory investigators were blinded to all clinical and culture data. NAAT results were independently classified as positive or negative for MTB by two investigators (JAK, CH), with a consensus reading reached after joint review of discrepant results. The secA1 and MTD data were unblinded only after a final assignment had been made for all samples.

### Statistical Analysis

We used STATA 11.0 (Stata Corporation, College Station, TX) for statistical analyses and defined a finding as significant if the probability of a two-tailed, type-I error (p-value) was <0.05. We calculated sensitivity, specificity, positive predictive value, and negative predictive value of each of the sputum NAATs in reference to two separate gold standards. For the primary analysis, we defined TB as present if a patient had ≥1 positive sputum or BAL mycobacterial culture. As a secondary analysis, we defined TB as present if cultures were positive or if a patient was clinically diagnosed with culture-negative TB at the 2-month follow-up visit. We planned in advance to compare diagnostic performance by HIV status using a two-sample test of proportions for unpaired samples. Assuming a TB prevalence of 30% and a sample size of 210 patients, we estimated that we would have 89% power to detect a 15% or larger increase in sensitivity for the secA1 NAAT compared to the MTD NAAT (previously reported as 76% in smear-negative patients), and 84% power to show that the specificity of the secA1 assay was within 5% of the previously reported value for the MTD assay of 97% [5]. Finally, we estimated the incremental value (additional sensitivity) of the two NAATs as add-on tests after smear microscopy [20] and measured the potential clinical impact on time-to-treatment initiation if a NAAT result were used to prescribe TB treatment, compared to clinical judgment alone [21].

## Results

### Participants

**Figure 1:**
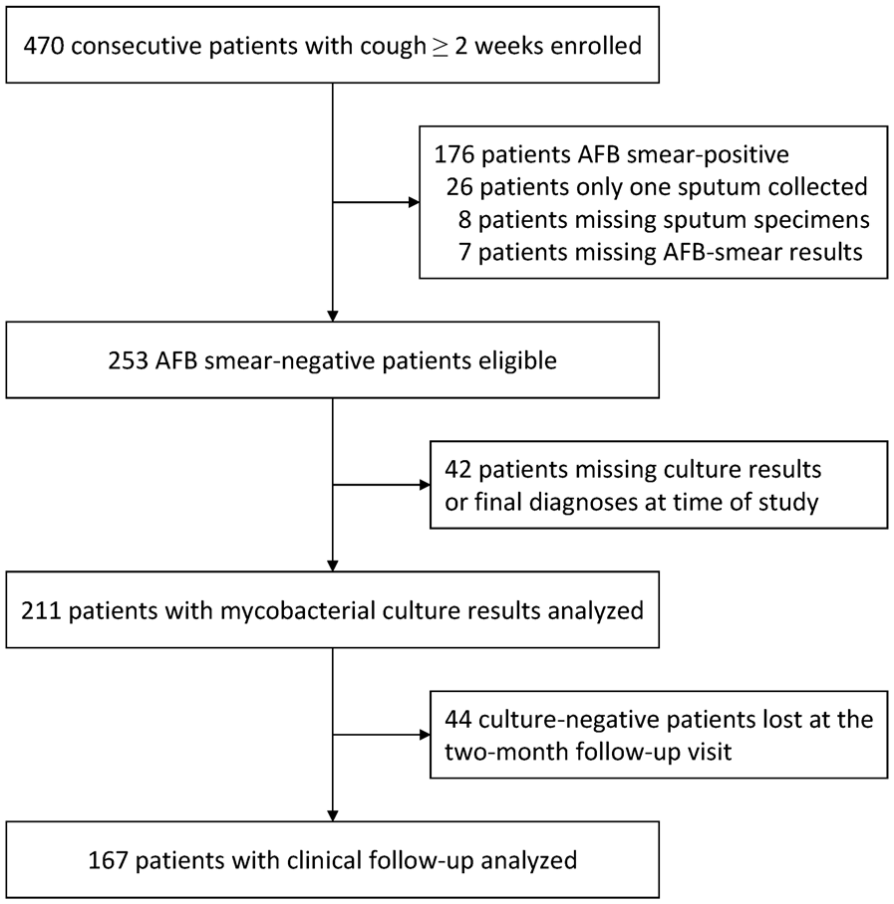
Patients enrolled, eligible, and analyzed.

Four hundred seventy consecutive adult patients with cough ≥2 weeks were enrolled in the overall study. One hundred seventy-six were sputum AFB smear-positive by Ziehl-Neelsen staining, which had a sensitivity of 66%; 26 had only one sputum collected; eight patients had missing sputum samples; and seven patients were missing AFB results. Of the remaining 253 patients, 42 were excluded because culture results or final diagnoses were unknown, leaving 211 patients for this analysis (Figure 1).

Patients had a median age of 35 years (inter-quartile range (IQR) 28–42; Table 1). Slightly more patients were female (114, 54%) than male, and the majority had received antibiotics prior to hospital admission (139, 66%). Most patients were HIV-seropositive (170, 81%), with a median CD4+ T-cell count of 78 cells/µL (IQR 29–203 cells/µL); 94 (55%) of the HIV-seropositive patients were taking PCP prophylaxis, and 29 (17%) were taking antiretroviral therapy. Patients with HIV tended to be younger (median age 34 years, IQR 28–39) than patients without HIV (median age 48 years, IQR 34–62; p<0.001), and more often female (57% vs. 41%; p = 0.072). Fifty-two of the 211 patients with negative sputum exams by conventional light microscopy had positive sputum smear examinations using concentrated fluorescence microscopy, but only 34 of these 52 patients (65%) had a confirmed TB diagnosis by mycobacterial culture or clinical follow-up. One hundred nine patients (52%) underwent bronchoscopy. No adverse events arose as a result of sputum collection or bronchoscopy. The clinical impacts of fluorescence microscopy and of bronchoscopy in this population have been previously described [9], [13].

**Table 1:** Demographic and clinical characteristics of the 211 study participants.

CharacteristicsN (%)*	All patients(n = 211)	HIV-seropositive(n = 170)	HIV-seronegative(n = 41)
Median age, years (IQR)	35 (28–42)	34 (28–39)	48 (34–62)
Women	114 (54)	97 (57)	17 (41)
Taking antibiotics prior to admission	139 (66)	115 (68)	24 (59)
Median CD4+ T-cell count, cells/µL (IQR)	- -	139 (29–203)	- -
Taking co-trimoxazole prophylaxis on admission	- -	94 (55)	- -
Taking antiretroviral therapy on admission	- -	29 (17)	- -
Culture-confirmed TB	75 (36)	67 (39)	8 (20)
Clinically confirmed or culture-confirmed TB	92 (55)†	81 (61)††	11 (32)†††
Two-month mortality	46 (24)§	41 (26)§§	5 (14)§§§

Abbreviations: IQR, inter-quartile range; N, number; TB, tuberculosis.

Legend:

*Characteristics presented as number and column percentage unless otherwise indicated. Missing or indeterminate results reduced the number at risk in the superscripted subsets as follows:

^†^N = 167;

^††^N = 133;

^†††^N = 34;

^§^N = 193;

^§§^N = 157;

^§§§^N = 36.

Seventy-five of the 211 patients (36%) had culture positive TB. An additional 17 patients who had negative mycobacterial cultures met clinical criteria for TB, while for 44 patients, an in-person clinical assessment could not be made because no follow-up evaluation occurred at two months. Thus, a total of 92 (55%) out of 167 patients had culture-confirmed or clinically confirmed TB. Among 193 patients with known vital status at two months, 46 (24%) had died, including 41 (26%) HIV-positive and 5 (14%) HIV-negative patients (mortality difference 12%, 95% CI -1 to +25, p = 0.13).

### Results of Sputum NAATs

Using mycobacterial culture results as the gold standard, the sensitivity of MTD for diagnosing TB was 39% (95% Confidence Interval (CI) 28–51), which was 15% higher (95% CI 2–27, p = 0.019) than that of secA1, which had a sensitivity of 24% (95% CI 15–35; Table 2). The specificities of MTD (95%, 95% CI 90–98) and secA1 (95%, 95% CI 90–98) were the same (specificity difference 0%, 95% CI −4.3 to +4.3, p = 1.0). The positive predictive value for MTD was 81% (95% CI 64–92), and the negative predictive value was 74% (95% CI 67–80). The secA1 had a positive predictive value of 72% (95% CI 51–88), and a negative predictive value of 69% (95% CI 62–76). One patient had an indeterminate secA1 result secondary to PCR inhibition that could not be overcome by dilution.

**Table 2:** Diagnostic performance of nucleic acid amplification tests for TB, compared to both the primary and secondary reference standards.

Index Test Results	Reference Positive(N)	Reference Negative(N)	Percent Sensitivity(95% CI)	Percent Specificity(95% CI)
Mycobacterial culture reference standard	75	136		
MTD				
Positive	29	7	39% (28–51)	95% (90–98)
Negative	46	129		
Indeterminate	-	-		
secA1			24% (15–35)	95% (90–98)
Positive	18	7		
Negative	57	128		
Indeterminate	-	1		
Clinical TB reference standard	92	75		
MTD			32% (22–42)	97% (91–100)
Positive	29	2		
Negative	63	73		
Indeterminate	-	-		
secA1			20% (12–29)	99% (93–100)
Positive	18	1		
Negative	74	74		
Indeterminate	-	-		

Abbreviations: CI, confidence interval; IQR, inter-quartile range; MTD, Gen-Probe Amplified® Mycobacterium Tuberculosis Direct test; secA1, secretory gene A1 target test; TB, tuberculosis.

When clinically-defined TB was used as the reference standard, both tests had slightly lower sensitivities but higher specificities. MTD had a sensitivity of 32% (95% CI 22–42) and secA1 a sensitivity of 20% (95% CI 12–29), with a sensitivity difference of 12% (95% CI 2–22, p = 0.019). Again, the specificity of MTD (97%, 95% CI 91–100) and secA1 (99%, 95% CI 93–100) were similar (specificity difference 1.3%, 95% CI −2.6 to +5.3, p = 1.0). The positive predictive value of MTD was 94% (95% CI 79-99), and the negative predictive value was 54% (95% CI 45–62). The secA1 had a positive predictive value of 95% (95% CI 74–100) and a negative predictive value of 50% (95% CI 42–58).

Both the secA1 and the MTD tests had a higher sensitivity in HIV–seropositive patients than HIV-seronegative patients, using culture as the reference standard, although the precision of the estimates among HIV-seronegatives was limited by the small number of patients (n = 41, Table 3). MTD had a sensitivity of 42% (95% CI 30–55) among HIV-seropositive patients and a sensitivity of 13% (95% CI 0–53) among HIV-seronegative patients (difference in sensitivity, 29%, 95% CI −7 to +65, p = 0.14). The secA1 had a sensitivity of 25% (95% CI 16–38) among HIV-seropositive patients, and 13% (95% CI 0–53) among HIV-seronegative patients (difference in sensitivity, 13%, 95% CI −19 to +44, p = 0.67). There were no major differences in sputum quality, volume, smear-grade, or culture-grade between patients with and without HIV (data not shown).

**Table 3:** Diagnostic performance of nucleic acid amplification tests for TB stratified by HIV status, compared to the primary reference standard only.

Index Test Results	Reference Positive(N)	Reference Negative(N)	Percent Sensitivity(95% CI)	Percent Specificity(95% CI)
Mycobacterial culture reference standard	75	136		
HIV-seropositive patients	67	103		
MTD			42% (30–55)	93% (87–97)
Positive	28	7		
Negative	39	96		
Indeterminate	-	-		
secA1			25% (16–38)	93% (86–97)
Positive	17	7		
Negative	50	95		
Indeterminate	-	1		
HIV-seronegative patients	8	33		
MTD			13% (0–53)	100% (89–100)
Positive	1	0		
Negative	7	33		
Indeterminate	-	-		
secA1			13% (0–53)	100% (89–100)
Positive	1	0		
Negative	7	33		
Indeterminate	-	-		

Abbreviations: CI, confidence interval; IQR, inter-quartile range; MTD, Gen-Probe Amplified® Mycobacterium Tuberculosis Direct test; secA1, secretory gene A1 target test; TB, tuberculosis.

### Potential Clinical Impact of Nucleic Acid Amplification Testing

At hospital discharge, only 25 (33%) of 75 AFB smear-negative patients ultimately diagnosed with culture-positive TB had been prescribed TB treatment. Had results of the more sensitive of the two NAATs, the MTD, been made available to clinicians within 24 hours of sputum collection, 18 additional TB patients could have been correctly started on TB treatment, while only two patients with negative mycobacterial cultures would have been inappropriately started on treatment. Thus, availability of MTD results would have lead to an absolute increase in sensitivity for TB of 24%, and a relative increase of 72% in early TB case detection among the Ziehl-Neelsen AFB-smear-negative population (43 TB patients if MTD results had been available compared to 25 TB patients when MTD results were not available). Instead of waiting for culture results to initiate TB treatment, these patients could have been started a median of 27 days (range 15–42 days) earlier. Unfortunately, 10 of these 18 smear-negative, MTD-positive, culture-positive TB patients died a median of 8.5 days (range 4–13 days) after enrollment, and it is unknown whether earlier initiation of treatment for these patients would have improved their outcomes.

### NAAT Performance and Impact among Patients Smear-negative by Fluorescence Microscopy

Among 159 patients with negative fluorescence microscopy results, the MTD was 29% (95% CI 17–44) sensitive in reference to mycobacterial culture, while secA1 was only 8% sensitive (95% CI 2–20). Both NAATs were equally specific in fluorescence microscopy smear-negative patients (98%, 95% CI 94–100). Among 48 fluorescence smear-negative, mycobacterial culture-positive patients, 12 (25%) patients were empirically initiated on TB treatment. Had MTD results been available to clinicians, an additional 10 (21%) patients could have been prescribed treatment (22 TB patients if MTD results had been available compared to 10 TB patients when MTD results were not available), an 83% relative increase in the proportion of fluorescence microscopy smear-negative cases initiated on treatment.

## Discussion

Adoption of new technologies for TB diagnosis in highly endemic areas is a challenge because of the start-up and training costs. Yet, increased use of molecular tests for TB would permit more expeditious initiation of treatment and other measures to reduce TB transmission, which could eventually pay large dividends for global TB control [22], [23]. Although studies suggest that NAATs are cost-effective for TB diagnosis even in low-income countries [24], [25], this technology has been used to only a limited extent to evaluate smear-negative TB suspects in sub-Saharan Africa (Table 4). These studies have reported variable sensitivity depending on HIV prevalence, but have consistently shown that these assays can identify a substantial proportion of smear-negative TB patients. Delays in adopting NAATs for TB reflect ongoing operational and feasibility concerns in low-income settings [26], but a lack of information about the clinical impact and incremental value of these tests beyond the standard diagnostic algorithm of sputum smear microscopy and clinician judgment may also have contributed to the slow uptake of this technology [21], [27].

**Table 4:** Diagnostic performance of sputum nucleic acid amplification testing for TB diagnosis among smear-negative TB suspects in sub-Saharan Africa.

Location (Year)	Test	HIVPrevalence	PercentSensitivity(95% CI)	Reference
Zambia (2001)	IS6110 PCR	75%*	40 (25–57)	[26]
Zambia (2001)	Gen-Probe A-MTD	75%*	60 (43–75)	[26]
South Africa† (2010)	Cepheid GeneXpert MTB/RIF	66%*	73 (65–79)	[6]
Uganda (2009)	secA1 PCR	46%	100 (63–100)	[7]
Kenya (2004)	Roche Amplicor MTB	35%	82‡ (76–87)	[30]

Abbreviations: IS, insertion sequence; PCR, polymerase chain reaction.

Legend: Studies with 30 or more smear-negative patients from sub-Saharan Africa were included.

*Estimated from population-specific HIV prevalence rather than directly measured;

† Also includes specimens from Azerbaijan, India, and Peru;

‡ Sensitivity based on 3 PCR specimens.

In a prospective diagnostic cohort study of patients suspected of TB in a low-income country with a high prevalence of TB and HIV, we have shown that NAATs identify many smear-negative TB patients whom clinicians would otherwise fail to diagnose. Although the sensitivities of the NAATs evaluated in our study were modest compared to mycobacterial culture, we found that, if routinely applied, same day NAAT would have decreased time-to-treatment initiation in smear-negative TB patients by almost four weeks. Because smear-negative TB may account for up-to-half of all TB cases in sub-Saharan Africa [1], our data suggest that finding a way to put nucleic acid tests into everyday use could increase TB case detection in the region by 20% or more.

We undertook this study because prior data from a small cohort of smear-negative patients suggested that the secA1 assay might have a high sensitivity in smear-negative patients. In the current study, however, the in-house secA1 PCR assay was less sensitive than a commercial amplification assay at identifying MTB in AFB-smear-negative sputum, although both assays were equally specific. These results are likely explained by the fact that secA1 is a single-copy gene, whereas the MTD test targets rRNA, which is present in multiple copies per organism. Because the comparative advantage of the secA1 assay actually lies in its ability to distinguish multiple mycobacterial species [19], it may be better suited for use in lower TB incidence settings, where non-tuberculous mycobacterial pathogens are common and speciation is clinically important.

The sensitivity of the MTD test was also relatively low in our study and lower than that reported in previous studies in similar populations [26]. This may reflect the effects of freezing, storage, thawing and delayed processing on the integrity of nucleic acids (especially RNA) in the sputum specimens. More importantly and especially noteworthy for future demonstration studies of NAATs in sputum smear-negative populations, the lower sensitivity we observed could be explained by the higher sensitivity of sputum smear microscopy (33% in the previous study vs. 66% in our study) and mycobacterial culture (71% in the previous study vs. 94% in our study) used to define the eligible population [26]. Use of poor-quality microscopy to select eligible patients, or poor-quality cultures to define the reference standard are well-known biases in diagnostic studies [28], [29], biases which are best avoided entirely by also reporting measures of clinical impact. Thus, it is important that MTD detected a substantial proportion of untreated cases of smear-negative TB, whether TB was defined clinically or microbiologically, and whether smear-negativity was defined using conventional light or concentrated fluorescence microscopy.

There were some limitations to the study. First, NAATs were performed in a research laboratory far from the clinical site, which prevented assessment of the accuracy or feasibility of the tests in the routine operational setting, and may have resulted in a lower sensitivity for the reasons noted above. Second, for specimens that required dilution because of PCR inhibition and proved to be negative after 3-to-100 fold dilution using the secA1 assay, improved sensitivity may have been seen if we had been able to eliminate the inhibition and run the assay at higher concentrations. Third, although the sensitivities of the NAATs in this study were much higher among HIV-seropositive patients than among HIV-seronegative patients, we had insufficient power to determine whether these differences were statistically significant. This will be an important issue for future studies to address. Finally, neither of the reference standards used in this study included liquid mycobacterial culture, which might have provided higher, more accurate estimates of NAAT specificity and positive predictive value, and lower estimates of sensitivity and negative predictive value.

In summary, we carried out a prospective diagnostic cohort study to evaluate the accuracy and potential effectiveness of nucleic acid amplification testing for TB diagnosis in a hospitalized population with a high incidence of HIV, and found modest sensitivity, excellent specificity, and a large potential clinical impact. Future studies should identify feasible ways to implement nucleic acid testing and evaluate them with attention not only to their diagnostic accuracy but also to their clinical and public health impact.
